# Line-Up Image Position in Simultaneous and Sequential Line-Ups: The Effects of Age and Viewing Distance on Selection Patterns

**DOI:** 10.3389/fpsyg.2020.01349

**Published:** 2020-07-01

**Authors:** Thomas J. Nyman, Jan Antfolk, James Michael Lampinen, Julia Korkman, Pekka Santtila

**Affiliations:** ^1^Faculty of Arts, Psychology and Theology, Åbo Akademi University, Turku, Finland; ^2^Faculty of Arts and Sciences, New York University Shanghai, Shanghai, China; ^3^Department of Psychological Science, University of Arkansas, Fayetteville, AR, United States

**Keywords:** eyewitness, line-up, simultaneous, sequential, position effects, age, distance, facial encoding strength

## Abstract

It is known that children and older adults produce more false alarms in target absent line-ups and that weaker facial encoding increases choosing bias. However, there has been no investigation of how age or facial encoding strength impacts line-up position selections in either sequential or simultaneous line-ups. In the present study, we presented participants with four live targets (one by one) while manipulating sequential and simultaneous line-ups between participants and target present and target absent line-ups within participants. In order to investigate facial encoding strength, we presented the targets at distances between 5 and 110 m. Our main hypotheses were that children due to deficits with inhibition would be more biased toward indiscriminate selections in the first position of sequential line-ups compared with subsequent line-up positions and that first position selections would increase for all age groups as facial encoding became weaker. In simultaneous line-ups, we expected to find a top row bias. In our sample (*N* = 1,588 participants; 6–77 years), we found that younger children (6–11 years) and the oldest adults (60–77 years) showed a first position bias in sequential line-ups, and as facial encoding became weaker, all age groups (6–11, 12–17, 18–44, 45–59, and 60–77 years) showed an increased tendency to make first position selections. We also found a weak top row preference in simultaneous line-ups, which was moderated by age and increased distance. The main finding is that the results suggest that younger children and the oldest adults had a tendency toward a first position selection bias in sequential line-ups. Based on the combined results, we recommend caution when using sequential line-ups with younger children or older adults.

## Introduction

Photograph line-ups (i.e., photo arrays) are commonly used by the police to investigate whether or not their suspect matches an eyewitness’s memory of the perpetrator of a crime, and a line-up identification or rejection can be used as corroborating or exonerating evidence of a suspect’s guilt (Wells, 1993; Wells and Olson, 2002). Photograph line-ups are typically arrangements of either sequentially or simultaneously presented images, where individuals are asked to select an image or to reject the line-up when the target (i.e., “the criminal”) is either present or not. The advantages and disadvantages of either line-up type have been debated for many years (e.g., Wells et al., 2015), but study samples have predominantly been based on young adults, with less focus on children, adolescents, and older adults, and very few comparisons have been made between age groups (Erickson et al., 2015; Fitzgerald and Price, 2015). Children and older adults tend to make more choices (compared to adults) in target absent line-ups (Bartlett and Memon, 2007; Bartlett, 2014; Fitzgerald and Price, 2015). The choosing bias found in children and older adults has been attributed to a reliance on familiarity rather than recollection (Shing et al., 2010), but there may also be other causes, as children show deficits in inhibition (Fitzgerald and Price, 2015), while this is less apparent in older adults (Rey-Mermet and Gade, 2018).

Surprisingly, although there are age differences in line-up choosing biases, there appear to have been no systematic investigations of the differences between line-up position selection patterns between age groups. This is relevant because if, for example, a child eyewitness (who has low inhibition and may rely on familiarity) is presented with a sequential line-up, it may be that their choosing bias is skewed toward selecting the first line-up position. Additionally, recent findings show that weaker facial encoding increases choosing rates (Nyman et al., 2019b; Smith et al., 2019). Combined, these results may indicate that age and facial encoding strength have an interactive effect on line-up position selections, manifested by, for example, children showing an increased tendency toward selecting the first line-up position due to both weaker facial encoding and low inhibition combined.

An investigation of the interactive effects of age and facial encoding strength on line-up position selections is of practical importance because eyewitnesses can be of any age and may witness a crime in circumstances that reduce facial encoding strength, such as at a greater distance or in lower lighting, factors that can easily be ascertained in an actual case. A better understanding of these factors can prevent reliance on false positives (i.e., misidentifications) in legal decisions. This is vital considering that misidentifications have been found to play a significant part in erroneous convictions in post-conviction exoneration cases in the United States, and their role in other countries around the globe is being assessed (Innocence Project, 2018). Differences between age groups in selection patterns may not be confined to eyewitness line-ups, as choosing biases also manifest in arrays that are part of everyday settings, such as selecting items in department stores (Bar-Hillel, 2015). Here, we investigated the interactive effects of decreased facial encoding (i.e., increased viewing distance) and eyewitness age on line-up position selections in simultaneous and sequential line-ups.

### Position Effects and Line-Up Types

The two most common photograph line-up procedures are sequential and simultaneous line-ups, and researchers are continuously debating about the advantage of either (e.g., Wells et al., 2015). Some argue that sequential line-ups are superior and others that simultaneous line-ups are superior (e.g., Steblay et al., 2011; Palmer and Brewer, 2012; Gronlund et al., 2015; Wells et al., 2015; Wixted et al., 2016). Leaving aside the debate, the difference is that sequential line-ups are proposed as a method of inducing an absolute judgment (Lindsay and Wells, 1985) because each decision (select or reject) is in relation to one image at a time, whereas simultaneous line-ups are suggested to encourage relative judgments (Wells, 1984) because the line-up images can be compared with each other at the same time. Furthermore, the absolute judgments in sequential line-ups are thought to be more connected with recollection, while the relative judgments in simultaneous line-ups may be more associated with perceived familiarity (Meissner, 2005).

The basic structure of the two line-up types is that in sequential line-ups, it is common to present four, six, or eight images in sequential order, one image at a time, while in simultaneous line-ups, the images are presented in an array consisting of two rows of two, three, or four images each. In both sequential and simultaneous line-ups, it is also common experimental practice to manipulate the line-ups so that participants are either shown a target present line-up, where the image of the target (i.e., “the criminal”) is part of the line-up, or a target absent line-up where the image of the target has been replaced by a similar looking filler (i.e., a known innocent). The target present and absent manipulation mimics the real-life situation where the police apprehend a suspect and present the suspect in a line-up to an eyewitness, while not knowing if the suspect is the actual criminal (i.e., target present) or not (i.e., target absent). By investigating both target present and absent line-ups in lab-based studies, it is possible to estimate the likelihood of an eyewitness making the a true positive (i.e., identifying the perpetrator) or a false positive (i.e., identifying an innocent person) under various conditions.

In sequential line-ups, position or order effects have been shown to be moderated by the next-best alternative (i.e., filler image), that is, if the next-best alternative in the line-up precedes the actual target, then this reduces the likelihood of a correct identification (Clark and Davey, 2005). Individuals also tend to be more cautious (i.e., conservative) at the start of a sequential line-up and then become increasingly liberal toward the end of the line-up (Meisters et al., 2018). However, some findings suggest that the shift from conservative to liberal only occurs when participants are aware of the number of images in the line-up; if they are not aware of this, then, the “pressure” to select toward the end of the line-up does not increase (Horry et al., 2012). These results have found support in later studies (Carlson et al., 2016), but findings are not uniform, and position effects have also been found in line-ups where no information has been given (Carlson et al., 2016; Meisters et al., 2018). Overall, there is little agreement regarding position effects in sequential line-ups, and there is an ongoing debate regarding how these effects should be evaluated (e.g., Wilson et al., 2019).

In simultaneous line-ups, participants tend to avoid the edge positions (i.e., edge-aversion) (O’Connell and Synnott, 2009), or to select from the bottom row more often (Sporer, 1993), or to select from the top row more often (Palmer et al., 2017; Carlson et al., 2019). There are also studies that have found no position effects in simultaneous line-ups (Clark and Davey, 2005; Meisters et al., 2018). Interestingly, outside of eyewitness research, simultaneous arrays can either produce edge aversion or an edge advantage, depending on how salient, representative, or focal the position of an item is in relation to a person’s reach in, for example, a department store (Bar-Hillel, 2015). The reasons for the aversion or the advantage are associated with decisions in relation to the task at hand, such as selecting a product in a shopping aisle, and may be influenced by more automatic habits such as scanning text or images from left to right (at least for a Western population), creating a left side edge bias (Bar-Hillel, 2015). However, in the case of a line-up task in eyewitness research, where the goal is to select an image based on the person’s memory, eye-tracking results indicate that left to right strategies only play a small role (Megreya et al., 2012). Furthermore, eye-tracking results show that individuals do not attend equally to all images in a line-up, indicating that the array structure has an impact (Mansour et al., 2009). In the present study, we investigated these possible differences by looking at top versus bottom row choosing bias and central versus peripheral position bias.

In general, there is little consistency within eyewitness research regarding reporting image position details or systematically investigating position effects, and this appears to have been a problem for a long time (cf. Memon and Gabbert, 2003). On average, reports indicate that positions are either counterbalanced or fixed, yet little detail is typically given, and some studies do not report line-up positions at all. This makes cross-study comparisons difficult.

### Position Effects and Eyewitness Age

To the best of the authors’ knowledge, no studies have explicitly investigated age differences in position effects. For example, some have investigated line-up accuracy in children and also (but less so) in adolescents and older adults, but the most recent meta-analysis regarding age differences in the eyewitness literature by Fitzgerald and Price (2015) makes no mention of line-up position effects. Concerning children, the only studies that relate to eyewitness age and position effects are those that deal with introducing a so-called “wildcard” (i.e., an image of a silhouette of a person with a question mark centered in the middle) or an elimination round (i.e., to eliminate all but one line-up member) in simultaneous line-ups, which are both used to increase the likelihood of a child making a correct target absent line-up rejection (Pozzulo and Lindsay, 1999; Zajac and Karageorge, 2009; Pozzulo et al., 2016; Sheahan et al., 2017). These manipulations have been attempts to combat children being prone to making a selection in target absent line-ups, which may be associated with low inhibition, social demands, or that children’s face processing strategies, compared with adults, are less effective (Fitzgerald and Price, 2015). From a developmental perspective, face perception appears to mature as early as between 5 and 7 years of age, and age-related differences in recognition accuracy may, therefore, be due to cognitive developments in attention and memory rather the face perception (Crookes and McKone, 2009).

Concerning adolescents, even less is known regarding both accuracy and position effects than there is regarding children (cf. Fitzgerald and Price, 2015). It is also unclear at exactly what age the choosing bias found in children dissipates, as it is not prevalent in young adults and does not always differ between older adolescents and young adults (Sheahan et al., 2017). However, findings on inhibition suggest that response inhibition develops into adulthood (Nigg, 2000; Fitzgerald and Price, 2015), indicating that also adolescents may show disinhibition similar to children. An interesting finding is that adolescents, in comparison with other age groups, appear to be more susceptible to selecting an innocent bystander in target absent line-ups, a result that has been interpreted to be the result of the developmental stages of progressing from childhood to adulthood (Brackmann et al., 2019).

At the upper end of the age spectrum, older adults sometimes show a similar choosing bias as children and to produce more false positives in target absent line-ups (Bartlett and Memon, 2007; Bartlett, 2014; Fitzgerald and Price, 2015). However, for older adults, it is less clear that disinhibition is an underlying cause (cf. Rey-Mermet and Gade, 2018), and although older adults may also yield to social pressure, there may be other causes as well, such as memory and cognitive decline due to aging (Fitzgerald and Price, 2015). An example of the differences is that children tend to be more accurate when they receive unbiased instructions (Rose et al., 2005; Pozzulo and Dempsey, 2006) and when there is low social pressure (Lowenstein et al., 2010), while these factors do not improve the performance of older adults, suggesting that memory deficits are at play in older adults (Shing et al., 2008). The differences and similarities between children, adolescents, and older adults, and the reasons for these differences are, nevertheless, still not completely understood as few studies have directly compared all these age groups simultaneously (Erickson et al., 2015; Fitzgerald and Price, 2015).

Although the earlier findings reviewed above do not relate directly to position effects, they, nevertheless, indicate both that selection patterns may differ between age groups and that age groups may also differ regarding what influences their decisions. An additional and important hypothesis regarding the choosing bias in children and older adults is that it may be the result of reliance on associative processing (i.e., familiarity) instead of strategic processing (i.e., recollection) (Shing et al., 2008, 2010). For a discussion regarding familiarity and recollection, see Jacoby (1991). The associative process of familiarity develops at a young age, whereas recollection continues to develop throughout childhood (Anooshian, 1999; Brainerd et al., 2004), and it may be that children depending on familiarity rather than recollection can explain their choosing bias (Shing et al., 2008, 2010). Older adults are purported to rely on familiarity rather than recollection due to recollection impairments (Healy et al., 2005; Fitzgerald and Price, 2015). In other words, it may be that for children and older adults (and perhaps also for young adolescents) the more liberal choosing bias is observed because more images seem familiar and are, so to speak, “good enough” (i.e., most familiar).

As there are age differences in selection patterns, then there are also testable hypotheses. For example, in a sequential line-up, the liberal response bias found in children and older adults would be expected to result in an increase in first position selections because any image shown would cross the threshold of the decision criterion (cf. Wilson et al., 2019). However, one difference between children and older adults is the lack of inhibitory control in the case of children, which might further result in a bias toward selecting the first position. This would manifest itself through children showing a more liberal bias in the first line-up position versus later positions. As older adults also rely on familiarity but have no inhibition deficits, this might lead to a choosing bias that is more similar across line-up positions (i.e., just as liberal). In simultaneous line-ups, it is less clear how processing based on familiarity and impulse control might reveal themselves making it more difficult to formulate specific hypotheses regarding these line-ups. Nevertheless, adults tend to avoid the edges (i.e., edge-aversion), and this is modulated by line-up instructions and the line-up shape (Palmer et al., 2017). This indicates that it is also important to investigate if there are differences in selection patterns between age groups, as the tendencies may vary in how instructions are understood and how different age groups react to the line-up array.

### Position Effects and Increased Distance

Increased distance has a negative impact on eyewitness accuracy (Jong et al., 2005; Loftus and Harley, 2005; Lindsay et al., 2008; Lampinen et al., 2014), and although the effect is similar for all age groups, the accuracy levels at close distances (i.e., 5 m) differ so that children and older adults are less accurate compared with young adults (Nyman et al., 2019b). Importantly, increased distance can be seen as a proxy for facial encoding strength because increased distance decreases the visual angle and the resolution of the face (Nyman et al., 2019a, b). In an earlier paper, we hypothesized that if differences in accuracy between age groups can be attributed to reliance on either familiarity or recollection, then increased distance should result in a higher degree of choosing for those that rely on familiarity (e.g., children and older adults) and less for those that rely on recollection (e.g., adults) (Nyman et al., 2019b). This assumption was based on the idea that when facial encoding strength is lower, then perhaps more faces will give rise to a familiarity effect, while for those relying on recollection, there will be fewer faces that give rise to a strong enough memory match. However, the findings of our previous study, on the same dataset, illustrated that increased viewing distance resulted in the adoption of a more liberal response criterion for all age groups and in both simultaneous and sequential line-ups (Nyman et al., 2019b). The findings that low facial encoding strength leads to an increase in choosing bias has also been corroborated in an earlier study (Mansour et al., 2012) and a more recent study (Smith et al., 2019).

These findings may also reflect a metacognitive error for all age groups, in that they failed to estimate increased viewing distance as a measure of the task difficulty, which rationally would have led to more line-up rejections. Instead, individuals became increasingly willing to select or to guess. Similar results reflecting an inability to metacognitively assess the difficulty of the task have been found in other studies (Mansour et al., 2012). Moreover, the tendency to choose despite the increasing task difficulty has been explained as a wish not to miss the opportunity of “getting the right person” (Smith et al., 2018).

Here, we were specifically interested in investigating if an increased liberal response criterion due to increased distance resulted in more first image selections in sequential line-ups for all age groups. Here, we assumed that younger children and older adults would make more first position selections compared with other age groups. We were also interested to investigate the possibility that young children (and perhaps adolescents), in contrast to older adults, would adopt a more liberal response bias in the first position compared to subsequent positions. This behavior might be the result of lower inhibitory control, as separate from a more liberal response criterion. In simultaneous line-ups, we had less precise hypotheses regarding the interactive effects of age and facial encoding strength on position effects, and we, therefore, ran exploratory analyses to investigate possible differences.

### The Present Study

In the present study, we investigated participants’ line-up position selections in sequential and simultaneous target present or target absent line-ups. The data were collected in an experimental setting, already reported in an earlier publication (Nyman et al., 2019b), where unfamiliar live targets were presented to participants at distances between 5 and 110 meters (m). Here, each participant was shown, in sequential order, four live targets that were each followed by an immediate line-up task. Although using multiple line-up designs is not as common as single line-up designs, this is a cost-effective approach that does not negatively impact eyewitness decisions (Mansour et al., 2017). The setup enabled us to investigate selection patterns and strategies under optimal conditions (i.e., clear viewing conditions, optimal duration, and an immediate line-up task) while manipulating the facial encoding strength by presenting live targets at varying distances. Additionally, due to our large sample size (*N* = 1588) and age range (6–77), we were also able to investigate differences between age groups. Based on the background literature, we formulated the following hypotheses:

(i) Based on the assumption that young children and adolescents have inhibition deficits, we expected that they would make more indiscriminate selections in first line-up positions in sequential line-ups compared with the other age groups. That is, these two age groups would be biased toward making more mistakes in the first line-up compared with other age groups (and compared with decisions in later line-up positions).
(ii) Based on the assumption that increased choosing bias is associated with weaker facial encoding strength (i.e., increased distance), we expected to find that with increased distance, there would be an increased tendency to select first line-up positions in sequential line-ups in all age groups. That is, as the criterion became more liberal, the number of first position selections would increase more than the number of selections in subsequent line-up positions. However, we also expected that the tendency would be stronger in young children, adolescents, and the oldest adults. Here, we also expected that children would show the highest level of first position selections because they may also have inhibition deficits, whereas older adults perhaps do not.
(iii) Based on the most recent findings on choosing bias in simultaneous line-ups (Palmer et al., 2017; Carlson et al., 2019), we expected to find a top row bias.

As there were many open questions due to the limited background literature, we ran some exploratory analyses based on the following assumptions:

(i) We were interested in the predictive value that line-up positions have on the likelihood of correct identifications and false alarms (i.e., selecting an innocent). This was relevant because we wished to investigate how the shifting criterion due to increased distance would impact the ability to correctly identify the target or mistakenly identify an innocent suspect in the first versus subsequent line-up positions. If increased distance induces a more liberal criterion, the question remains how this affects accuracy in the line-up positions.
(ii) As a continuation of the investigation of line-up position accuracy, we also conducted a confidence accuracy characteristic CAC analysis to better understand the relationship between accuracy and postdictive confidence. This gives much relevant information regarding selection patterns and how the shifting criterion is impacting decisions.
(iii) As some findings have also found edge-aversion in simultaneous line-ups, we also wanted to investigate possible edge-aversion effects in children and adolescents. Here, we assumed that inhibitory deficits would lead to children and adolescents focusing on the most salient line-up positions (i.e., the central positions) and disregarding positions at the edges (i.e., peripheral positions).

## Materials and Methods

### Participants

The present dataset is comprised of 1,588 participants (see Table 1). The dataset was collected in connection with an earlier publication, and additional information can be found in our previous study (Nyman et al., 2019b). Each participant was presented with four live targets one at a time and took part in four line-up tasks immediately after having observed a target. The data are available at https://osf.io/bqdmg/.

**TABLE 1:** Distribution of participants by age group and gender.

Age	Description	*n*	Mean age	*SD*	Female	Male	Other
6–11	Young children	266	9.31	1.45	147	112	7
12–17	Older children	311	13.71	1.61	184	121	6
18–44	Young adults	690	31.95	7.49	436	237	17
45–59	Older adults	225	49.88	3.96	136	88	1
60–77	Oldest adults	96	67.13	5.48	58	30	8
Total	All participants	1588	29.25	17.13	961	588	39

We recruited participants on site and informed them of the experimental design before they took part. We gave no compensation for participation. If participants were 11 or younger, they could only participate if both a close relative to the child and the child, themselves, could give their verbal consent.

### Ethics Statement

All aspects of the current experimental design were approved by The Ethical Committee at Åbo Akademi University, and all aspects of the data collection prior to the current experiment were approved by the Ethical Committee at the Department of Psychology and Logopedics at Åbo Akademi University.

### Stimuli

We employed four ethnic Finnish targets: two female and two male targets. The targets were of average height and weight and were between 19 and 27 years old (*M* = 22.3, *SD* = 3.4).

### Measures

During the experiment, we presented all our questions and collected all answers on tablets. We had placed a tablet in front of each participant so that the participant could respond via the touch screen. All demographic information, line-up task images, and pre and post line-up questions were administered via the tablet (see section “Procedure” below). The identification and rejection accuracies have been presented in an earlier publication (Nyman et al., 2019b). In the current study, we present selection patterns based on how the participant selected images in the photo arrays. The photo arrays consisted of eight images. In both simultaneous and sequential target present line-ups, there was one target image and seven filler images, and in the target absent line-ups, the same seven fillers were used, and an eighth filler replaced the target photo. In simultaneous line-ups, the images were presented in two rows of four images, and in sequential line-ups, the images were presented one per page. In both line-ups, all images and positions were automatically randomized by the software, and all image sizes were kept constant. When analyzing the frequency distributions of target images, we found that our randomizations had been successful. Although many cells had small expected frequencies making the chi square tests potentially unreliable, we found that the distribution of target images per age group, distance, and line-up positions in target present sequential line-ups [χ^2^(2,530) = 2613.9, *p* = 0.120] and simultaneous line-ups [χ^2^(2,530) = 2,519.2, *p* = 0.606] were balanced. In sequential line-ups, all participants knew prior to the line-up that a total of eight images would be presented, unless an image was selected whereupon the line-up would end (i.e., there was an absolute stopping rule). The line-up creation has also been presented in more detail in an earlier publication (Nyman et al., 2019b).

### Procedure

All participants were fully informed about the experimental design prior to participation. Up to four participants could take part simultaneously (via individual tablets and separated by screens). The tablet followed the following structure: (1) participant’s demographic information, (2) visual acuity test, (3) test-round (for the sake of practice), (4) viewing the first target and conducting line-up task number 1, (5) target 2 and line-up task 2, (6) target 3 and line-up task 3, and (7) target 4 and line-up task 4. Targets were presented for 20 s, and each line-up task was conducted immediately after viewing a target. The line-up task consisted of estimating the distance to the target, the gender, age, weight, and height of the target, followed by a simultaneous or sequential array (manipulated between participants) that was either target present or target absent (manipulated within participants). In the present study, we do not report information regarding the estimates in relation to the targets or overall line-up accuracy. This information has been disseminated in an earlier work (Nyman et al., 2019b) and a work in progress.

### Design

We presented four live targets at one of 16 possible distances between 5 and 110 m. Between 5 and 50 m, the increase between distances was 5 m, and between 50 and 110 m, the increase between distance was 10 m. We also designed the randomization so that each of the four targets would be presented in one of four blocks: (1) 5–20 m; (2) 25–40 m; (3) 45–70 m; (4) 80–110 m. We did this in order to present all participants with varying degrees of difficulty. The setup was created outdoors, and the measured lighting during the experiment was 43,139 lx (*SD* = 33,452, *min* = 4,005 lx, *max* = 999,999 lx). In an earlier publication on the same dataset, we categorized age into four groups: young children: 6–11 years, older children: 12–17 years, young adults: 18–44 years, older adults: 45–77 years (Nyman et al., 2019b). In the present study, we have further split the last age group (45- to 77-year-olds) into two categories, older adults 45–59 years and the oldest adults 60–77 years, in order to better capture possible age-related issues. The new cut-offs were based on age categorizations in earlier studies (cf. Erickson et al., 2015).

### Statistical Analyses

#### Main Analyses

Our aim was to investigate the effects of age group, distance, presentation order (i.e., trial), line-up type, and line-up position, on image selections, and due to the complex and nested nature of our design, we used multilevel logistic regressions to analyze the data. A multilevel logistic regression provides a log odds value that can be used to calculate the probability of a response being correct. The estimates that we present in the text and in the figures are based on the predicted probabilities from the multilevel logistic regressions. All analyses were run in the platform R (R Core Team, 2016). For the logistic regression, we used the lme4 package (Bates et al., 2014), and the figures based on the outcomes from these models were created using the SjPlot package and ggplot2 package (Wickham, 2016; Lüdecke, 2019). Furthermore, in order to obtain main effects and interactions (Type-III sums of squares) for our predictors, we used the “afex” package (Singmann et al., 2019), and for *post hoc* comparisons, we used the package “emmeans” (Lenth et al., 2019). As we were interested in both specific comparisons in line with our hypotheses and exploratory comparisons, we opted for *post hoc* tests and present only the most relevant comparisons in the main text (and we only report the values for the significant results). The *post hoc* comparisons were used to investigate the differences within and between age groups at the four distance blocks (i.e., 5–20, 25–40, 45–70, and 80–110 m). That is, in contrast to our main analyses where distance was treated as a continuous predictor, in the *post hoc* analyses, we treated distance as a factor with four levels in order to compare between smaller distances (5–20 m) with greater distance (80–110 m). We did this to investigate how the effect of increased distance was moderated by the other predictors in the interactions. Regarding age groups, we were specifically interested in comparing how other age groups compared with our reference group (i.e., 18- to 44-year-olds) and have, therefore, focused primarily on these comparisons. Furthermore, in line with our hypotheses concerning inhibition and memory strength, we were especially interested in comparing age differences in first position sequential line-up selections at smaller and greater distances. Prior to running our analyses, we dichotomized the trial order into first versus second to fourth to investigate the difference between instances were an eyewitness only takes part in one line-up task (as is often the case in real life) or takes part in multiple line-up tasks (as in lab-based studies). This dichotomization was also conducted in a previous study on the same dataset, where we found no effect on identification or rejection accuracy (Nyman et al., 2019b).

In sequential line-ups, we also dichotomized the positions to first versus second to eighth, in accordance with our hypotheses. We then initially ran a complete model where selection was the outcome variable, and the predictors were distance, age, target present or absent, trial order, and line-up position. This complex model revealed that there was no effect of trial, so we decided to exclude trials as a factor in our next and simplified analysis. We also decided to analyze the target present and target absent data separately, in order to better be able to interpret the complex interactions. We included the targets as a random effect in order to generalize across all four targets. We did not include participants as a random effect because the model indicated that adding this effect did not add anything to the model. This resulted in two models where the outcome was selection and the predictors were distance, age group, positions, and targets were included as random effects.

In simultaneous line-ups, we also dichotomized positions into either the top versus bottom row (four images in each) in accordance with our main hypothesis. In simultaneous line-ups, we otherwise used the same analytical approach as in the sequential line-ups.

#### Exploratory Analyses

As we were also interested in exploring differences between central and peripheral choosing bias in simultaneous line-ups, we also dichotomized positions into either central or peripheral images (four images in both: the two middle images from the top and bottom row, and the two peripheral images from the top and the bottom row). We then ran the same analysis as when investigating top versus bottom row biases, by substituting the dichotomized predictor top versus bottom with central versus peripheral position selection.

Next, as we were interested in the predictive value of line-up positions in both sequential and simultaneous line-ups, we conducted exploratory analyses of the likelihood of a correct identification in target present line-ups and the likelihood of a false alarm (i.e., innocent selection) in target absent line-ups. As we had already investigated the effects of age and distance on these outcomes earlier (Nyman et al., 2019b), we here focused on the effects of line-up position by adding, in the analyses of sequential line-ups, the dichotomized choice of line-up position 1 or selecting line-up positions 2–8, and in the analyses of simultaneous line-ups, the dichotomized choice of top or bottom (and central and peripheral) line-up positions. To do this, we investigated the effects of distance, age, and the dichotomized line-up position choice on the likelihood of making a correct target present identification in sequential or simultaneous line-ups. We ran a multilevel logistic regression where the outcome variable was correct or incorrect identification, and the predictors were distance, age, and dichotomized line-up position choice. This gave us the likelihood of an accurate response (i.e., the hit rate) in both line-ups. Second, we wished to investigate the effects of distance, age, and the dichotomized line-up position choice on the likelihood of incorrectly selecting an innocent suspect in target absent line-ups (i.e., the false alarm rate). Here, the innocent subject was defined as the four most selected fillers (per line-up) in the dataset. This was also important as earlier results show that individuals have a tendency to shift to selecting the most similar image in target absent line-ups, a so-called target-to-foil-shift from target present to target absent line-ups (Clark and Davey, 2005). By using selection-based innocent suspects to calculate false alarm rates, we could capture the target-to-foil-shift effect. We used the same model to analyze false alarms as we did to analyze hit rates.

After having investigated the relationship between hit and false alarm rates per line-up position, we next turned to the relationship between confidence and accuracy per line-up position. To achieve this, we conducted separate confidence accuracy characteristic (CAC) analyses (Mickes, 2015) per age group and line-up position. In eyewitness research, confidence is often used as a postdictive measure of accuracy because it has been found that higher confidence is associated with accuracy and is an indicator that a photo in a line-up has elicited a strong memory match. However, if an individual has a liberal response criterion, then, we can assume that they will make identifications even when confidence is low, and when the response criterion is conservative, this should mean they only make an identification when they are very certain. In the present dataset, we already know that the response criterion is more conservative at closer distances compared with greater distance. That is, when the viewing distance was greater, participants of all age groups tended to guess more. A CAC analyses per line-up position helps investigate if there could have been differences in response bias between line-up positions. We categorized post line-up confidence into three categories, 0–60, 61–90, and 91–100%, which is an often used approach (e.g., Mickes, 2015). Furthermore, as we were mainly interested in the confidence–accuracy relationship between first versus subsequent positions in sequential line-ups, we also collapsed the line-up positions 2–8 into one category.

## Results

### Raw Data Overview

**FIGURE 1:**
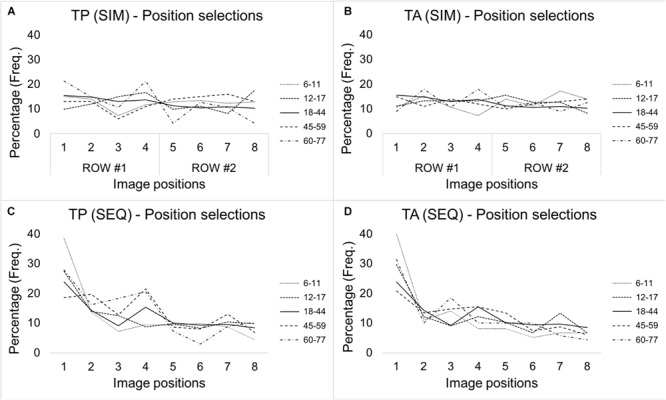
Illustrations of the percentages of images selected per line-up position, line-up type, and age group. **(A)** Upper left: The percentages of selected images per line-up position and age group in target present simultaneous line-ups and **(B)** upper right: the same in target absent line-ups. **(C)** Bottom left: the percentages of selected images per line-up position and age group in target present sequential line-ups and **(D)** bottom right: the same in target absent line-ups.

We have broken down the frequencies of observed target identifications, filler identifications, and rejections per distance block (i.e., 5–20, 25–40, 45–70, and 80–110), per age group (6–11, 12–17, 18–44, 45–59, and 60–77), and line-up type (sequential, simultaneous; target present, target absent). These frequencies can be found in Supplementary Materials A. To summarize the data, we have also illustrated the overall percentages of selections per line-up position (collapsed across all distances) in Figure 1. Based on a visual inspection of Figure 1, it appears that there is a tendency for all age groups to select the first position in sequential line-ups, whereas patterns in simultaneous line-ups are more difficult to interpret.

We have also collated the frequencies (collapsed across distance and trials) of the number of participants who selected the first line-up position or line-up positions 2–8 (combined), and the number of participants who rejected the line-up once, twice, three, or four times (see Table 2). From Table 2, it can be seen that it was rare for the participants to select position one in all four trials. The majority of the participants selected the first position only once or twice, and in a similar manner, the majority rejected the line-ups only once or twice. Importantly, the target present and absent line-ups were randomized per trial across all participants, not balanced (50/50) within the four trials each participant took part in, meaning that one participant can have been presented with, for example, four target absent line-ups. This means that rejecting multiple line-ups may have been an adequate response-strategy. However, as the image positions were randomized per trial, the likelihood of the target being in the first position repeatedly between trials for one participant is small. In other words, repeatedly selecting the first image is less likely to reflect a strategy based on facial encoding strength.

**TABLE 2:** Frequencies of image selections in sequential line-ups per age group and participants who selected or rejected the line-up one to four times.

Age group	Frequency of first position selections	Frequency of participants who selected first position 1, 2, 3, 4 times				Frequency of 2–8 position selections	Frequency of rejections	Frequency of participants who rejected 1, 2, 3, 4 times			
		1	2	3	4			1	2	3	4
6–11	116	39	20	7	4	180	209	37	38	20	9
12–17	102	47	14	5	3	256	216	55	54	15	2
18–44	178	106	27	2	3	621	581	122	117	59	12
45–59	61	41	7	2	0	250	165	50	32	13	3
60–77	41	18	8	1	1	97	60	13	9	7	2

*The complete dataset contains 6,233 observations for simultaneous and sequential line-ups. This table contains only the subset of responses for sequential line-ups, which is 3,133 observations. This sequential subset contains 498 first position selections, 1,404 2–8 position selections, and 1,231 line-up rejections.*

### Line-Up Position Selections

#### Target Present Sequential Line-Ups

**FIGURE 2:**
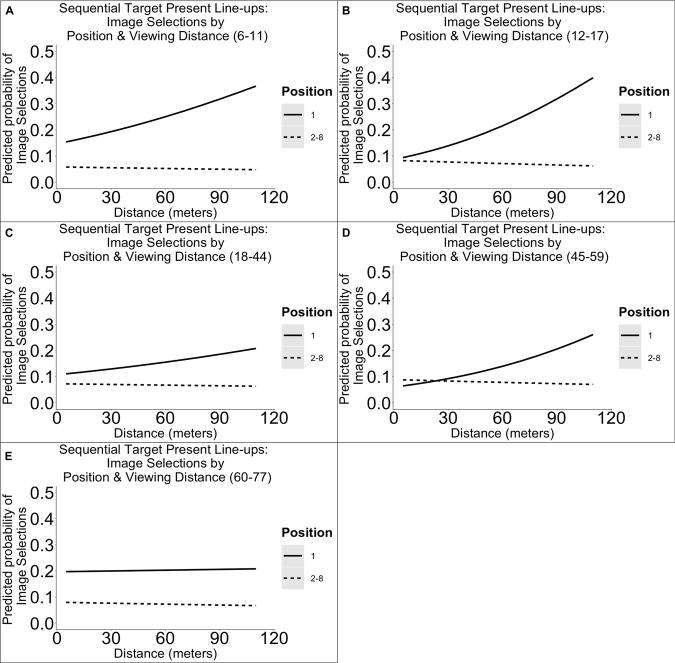
Illustration of the predicted probabilities of image selections in sequential target present line-ups per dichotomized line-up position, distance, and age group. Each panel represents the probabilities for a single age group: 6–11 **(A)**, 12–17 **(B)**, 18–44 **(C)**, 45–59 **(D)**, 60–77 years **(E)**.

The results from our analyses of target present sequential line-ups revealed that there was a main effect of distance [χ^2^(1) = 8.44, *p* = 0.004] and position [χ^2^(1) = 5.44, *p* = 0.020], an interactive effect between distance and position [χ^2^(1) = 18.64, *p* < 0.001], and an interactive effect between age and position [χ^2^(4) = 9.55, *p* = 0.049] on image selections. We have plotted the estimated outcomes in Figure 2. *Post hoc* results revealed that at smaller distances, the likelihood of making a selection in the first position did not differ from positions 2–8. At greater distances, in comparison with the first position, the likelihood of making a selection was lower for positions 2–8 (*B* = 1.59, *SE* = 0.17, *p* < 0.001). *Post hoc* results revealed that in comparison with 18- to 44-year-olds, 6- to 11-year-olds (but not other age groups) were more likely to select the first position (*B* = −0.54, *SE* = 0.19, *p* = 0.004) and less likely of selecting positions 2–8 (*B* = 0.26, *SE* = 0.12, *p* = 0.030). Importantly, when interpreting the results in Figure 2, the base rate probability (assuming all positions are equal) is 0.125 (i.e., 1/8) for both the first position and positions 2–8 (because the percentages for positions 2–8 were divided by 7). Here, we have assumed an equal distribution of selection percentages per cell as a baseline because we randomized the position of the target and filler images in each trial.

#### Target Absent Sequential Line-Ups

**FIGURE 3:**
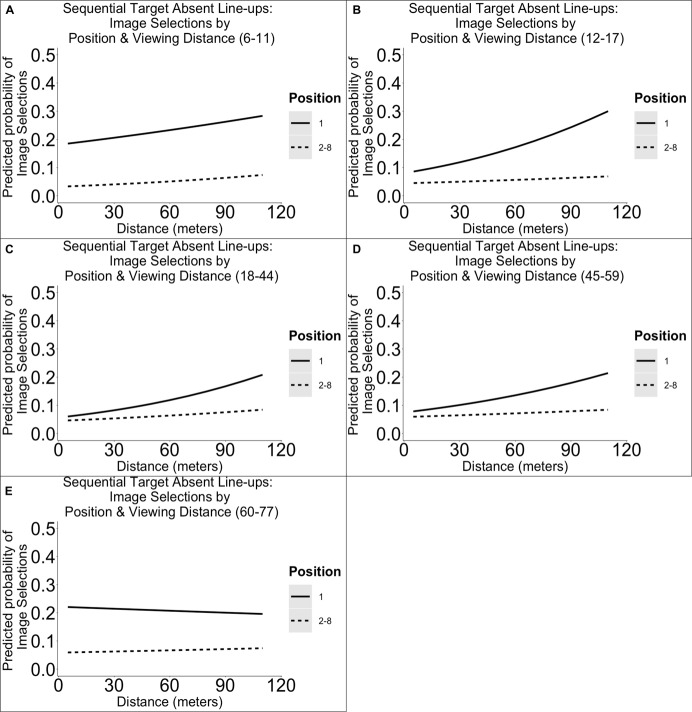
Illustration of the predicted probabilities of image selections in sequential target absent line-ups per dichotomized line-up position, distance, and age group. Each panel represents the probabilities for a single age group: 6–11 **(A)**, 12–17 **(B)**, 18–44 **(C)**, 45–59 **(D)**, 60–77 years **(E)**.

The results from our analyses of target absent sequential line-ups revealed that there was a main effect of distance [χ^2^(1) = 19.85, *p* < 0.001], age group [χ^2^(4) = 10.83, *p* = 0.029], and position [χ^2^(1) = 22.75, *p* < 0.001], and an interactive effect between age and position [χ^2^(4) = 16.04, *p* = 0.003] on image selections. We have plotted the estimated outcomes in Figure 3. The results show that as distance increased, so did the likelihood of selections and that the likelihood of making a selection was higher in the first position. *Post hoc* results also revealed that in comparison with 18- to 44-year-olds, 6- to 11-year-olds (*B* = −0.93, *SE* = 0.21, *p* < 0.001), 12- to 17-year-olds (*B* = −0.44, *SE* = 0.22, *p* = 0.042), and 60- to 77-year-olds (*B* = −0.84, *SE* = 0.28, *p* = 0.003), but not 45- to 59-year-olds, were more likely to select the first position, and 6- to 11-year-olds (*B* = 0.26, *SE* = 0.13, *p* = 0.048), but not other age groups, were less likely to select positions 2–8.

#### Target Present Simultaneous Line-Ups

**FIGURE 4:**
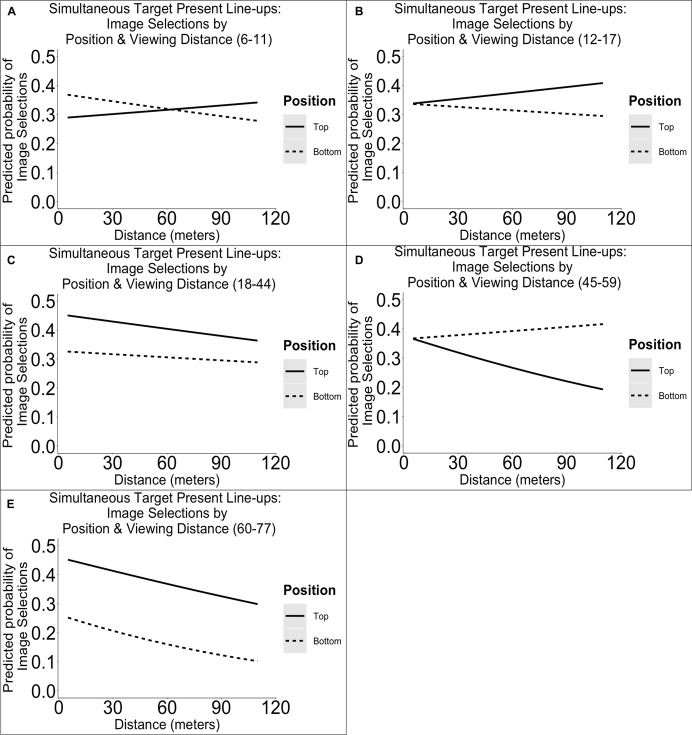
Illustration of the predicted probabilities of image selections in simultaneous target present line-ups per dichotomized line-up position (top versus bottom), distance, and age group. Each panel represents the probabilities for a single age group: 6–11 **(A)**, 12–17 **(B)**, 18–44 **(C)**, 45–59 **(D)**, 60–77 years **(E)**.

The results from our analyses of target present simultaneous line-ups showed no main effects of distance, age group, or top versus bottom line-up position, and no interactions on image selections. We have plotted the estimated outcome in Figure 4. Our exploratory analyses of central versus peripheral line-up position effects in target present simultaneous line-ups also revealed no main effects. We have plotted the outcome in Supplementary Figure S1 in Supplementary Materials B.

#### Target Absent Simultaneous Line-Ups

**FIGURE 5:**
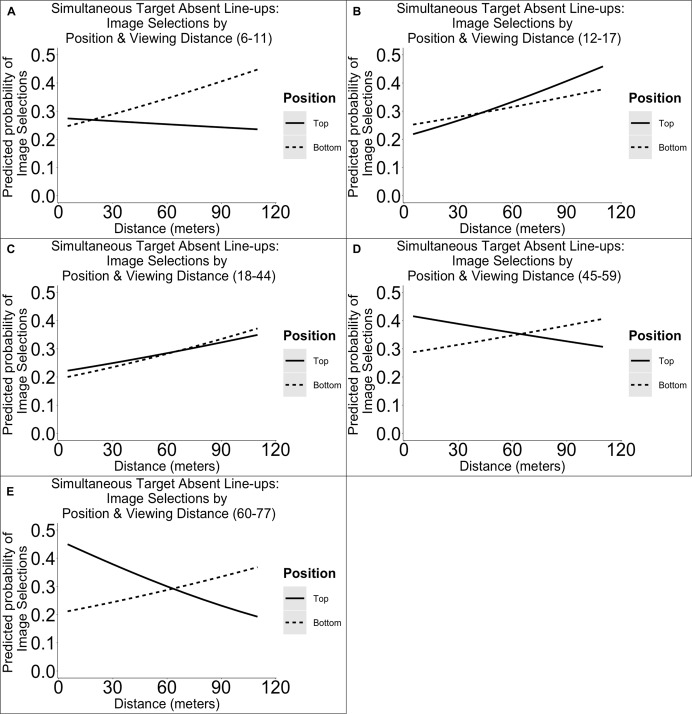
Illustration of the predicted probabilities of image selections in simultaneous target absent line-ups per dichotomized line-up position (top versus bottom), distance, and age group. Each panel represents the probabilities for a single age group: 6–11 **(A)**, 12–17 **(B)**, 18–44 **(C)**, 45–59 **(D)**, 60–77 years **(E)**.

In target absent simultaneous line-ups, we found a main effect distance [χ^2^(1) = 4.47, *p* = 0.034], age group [χ^2^(4) = 12.74, *p* = 0.013], top versus bottom row position [χ^2^(1) = 4.61, *p* = 0.034], and an interaction between distance and position [χ^2^(1) = 5.07, *p* = 0.019] on image selections. We have plotted the estimated outcomes in Figure 5. *Post hoc* comparisons revealed that compared to 18- to 44-year-olds, 45- to 59-year-olds (but not other age group) were more likely to make selections (*B* = −0.40, *SE* = 0.12, *p* < 0.001). Owing to the categorization of top or bottom row, the expected base rate was 0.5 (i.e., 1/2). *Post hoc* results indicate that position selections varied as a function of facial encoding strength, so that in some cases, there was a top row preference, and in other cases, there was a bottom row preference. These results are difficult to interpret but suggest that top or bottom row bias is moderated by encoding strength and, based on the figures, also by age.

Next, our exploratory analyses of central versus peripheral line-up position effects in target absent simultaneous line-ups revealed a main effect distance [χ^2^(1) = 4.36, *p* = 0.037], age group [χ^2^(4) = 13.13, *p* = 0.011], an interaction between age and position [χ^2^(4) = 10.57, *p* = 0.032], and an interaction between distance, age group, and position [χ^2^(4) = 12.52, *p* = 0.014] on image selections. We have plotted the outcome in Supplementary Figure S2 in Supplementary Materials B. *Post hoc* comparisons revealed that compared with smaller distances (Block 1: 5–20 m), the likelihood of making a central line-up position selection was higher at a greater distance (Block 4: 80–110 m) for 12- to 17-year-olds (*B* = −1.18, *SE* = 0.37, *p* = 0.002) and 18- to 44-year-olds (*B* = −0.55, *SE* = 0.26, *p* = 0.038), but not other age groups. *Post hoc* comparisons also revealed that compared with smaller distances (Block 1: 5–20 m), the likelihood of making a peripheral line-up position selection was higher at a greater distance (Block 4: 80–110 m) for 6- to 11-year-olds (*B* = −1.02, *SE* = 0.44, *p* = 0.021) and 18- to 44-year-olds (*B* = −0.69, *SE* = 0.25, *p* = 0.006), but not other age groups. As can be seen in Supplementary Figure S2 of Supplementary Materials B, the results are difficult to interpret, but indicated that age groups differed in how they selected central or peripheral positions, so that with increased distance, the selection of central image increased for all age groups except for young children, where the likelihood instead decreased (see Supplementary Figure S2 Supplementary Materials B). Moreover, as distance increased, the likelihood of selecting peripheral images increased for all age groups except for older adults and the oldest adults, where the likelihood instead decreased (see Supplementary Figure S2 in Supplementary Materials B).

### Line-Up Position Effects on Hit and False Alarm Rates

#### Sequential Line-Ups

Next, we investigated the effects line-up position on hit rates in sequential line-ups. In target present line-ups, we found a main effect of distance [χ^2^(1) = 32.98, *p* < 0.001], position [χ^2^(1) = 10.37, *p* = 0.001], and an interactive effect between distance and age group [χ^2^(4) = 12.60, *p* = 0.013] on correct identifications. As the effects of distance and age group have been discussed in an earlier paper, we here focus on the difference between line-up positions. The outcomes have been plotted per age group in Supplementary Figure S3 in Supplementary Materials B, where it can be seen that, in comparison with the first line-up, the likelihood of a correct response was higher in positions 2–8 (*B* = −0.72, *SE* = 0.26, *p* = 0.005). This illustrates that accuracy was lower when selecting an image in the first position. Next, investigating the effects line-up position has on false alarm rates in sequential line-ups, we found no main effects or interactions. The results are plotted in Supplementary Figure S4 in Supplementary Materials B. The results suggest that selections in either the first line-up position or positions 2–8 did not differ substantially in their likelihood of selecting an innocent suspect. Combined, this shows that, whereas false alarms were relatively constant, hit rates were better in positions 2–8, and this means that overall diagnosticity was better in later positions.

#### Simultaneous Line-Ups

Investigating the predictive value of line-up positions (top versus bottom row) in target present simultaneous line-ups, we found that there was a main effect of distance [χ^2^(1) = 126.79, *p* < 0.001], of position [χ^2^(1) = 7.93, *p* = 0.005], and an interactive effect between distance and age [χ^2^(4) = 9.96, *p* = 0.041] on correct identifications. The results have been plotted in Supplementary Figure S5 in Supplementary Materials B, and a *post hoc* test confirmed that in comparison with the top row, the likelihood of a correct response was lower in the bottom row (*B* = 1.31, *SE* = 0.29, *p* < 0.001). The results from our analyses of false alarm rates in target absent line-ups gave rise to a main effect of distance [χ^2^(1) = 4.66, *p* = 0.031] and of position [χ^2^(1) = 26.38, *p* < 0.001] on innocent identifications. The results have been plotted in Supplementary Figure S6 in Supplementary Materials B. *Post hoc* comparisons revealed that in comparison with the top row, the likelihood of an innocent identification was lower in the bottom row (*B* = 1.16, *SE* = 0.18, *p* < 0.001). Combined, this indicates that there was no substantial difference in diagnosticity between the top and bottom rows.

Next, looking at central and peripheral effects, we found a main effect of distance [χ^2^(1) = 112.57, *p* < 0.001] and of position [χ^2^(1) = 13.63, *p* < 0.001] on correct identifications. We have plotted the results in Supplementary Figure S7 in Supplementary Materials B. *Post hoc* comparisons revealed that in comparison with the central positions, the likelihood of a false alarm was lower in the peripheral positions (*B* = 1.16, *SE* = 0.18, *p* < 0.001). The results from our analyses of false alarm rates in target absent line-ups also revealed main effects of distance [χ^2^(1) = 8.14, *p* = 0.004] and of position [χ^2^(1) = 13.38, *p* < 0.001] on innocent identifications. We have also plotted these results in Supplementary Figure S8 in Supplementary Materials B. *Post hoc* comparisons revealed that compared with the central positions, the likelihood of an innocent identification was lower in the peripheral positions (*B* = 1.29, *SE* = 0.19, *p* < 0.001). Combined, this indicates that there was no substantial difference in diagnosticity between central and peripheral positions.

#### Confidence Accuracy Characteristic

To give an overview of the target present target selections and target absent filler selections used to calculate the CAC percentages, we have created a table with the selection frequencies per age group and line-up position in sequential line-ups (see Table 3). Based on these frequencies, we have calculated the CAC percentages per line-up position and have presented them in Table 4. However, as we were mainly interested in comparing the first line-up position with line-up positions 2–8, we have also presented the selection frequencies in line-up position 1 versus 2–8 (see Table 5) and then the CAC percentages based on these frequencies in Table 6.

**TABLE 3:** Frequencies of TP target and TA filler selections in sequential line-ups per position and confidence category.

Age group	Selection	*N*	Position 1			Position 2			Position 3			Position 4			Position 5			Position 6			Position 7			Position 8		
			0–60	61–90	91–100	0–60	61–90	91–100	0–60	61–90	91–100	0–60	61–90	91–100	0–60	61–90	91–100	0–60	61–90	91–100	0–60	61-90	91–100	0–60	61–90	91–100
6–11	TP target	42	3	4	2	4	0	3	2	0	1	2	0	0	0	3	0	3	5	0	5	2	2	0	1	0
	TA filler	135	30	12	12	8	4	3	14	2	3	8	3	0	5	3	3	7	0	0	8	1	0	8	1	0
12–17	TP target	68	7	3	4	7	3	0	7	5	0	2	0	0	2	4	3	1	2	2	4	3	1	3	5	0
	TA filler	165	31	11	7	14	3	3	10	4	1	15	5	0	11	5	1	9	2	0	17	5	0	7	2	2
18–44	TP target	165	12	10	4	7	9	5	13	5	2	10	8	4	8	6	5	14	6	2	11	7	1	10	4	2
	TA filler	372	53	18	5	35	15	0	30	13	1	28	14	0	40	7	1	30	7	0	44	2	0	21	8	0
45–59	TP target	53	2	0	2	3	5	1	4	5	1	3	3	4	1	3	0	0	0	2	8	1	0	3	1	1
	TA filler	149	21	8	2	12	8	0	17	3	2	17	5	1	14	5	1	6	4	1	12	1	0	8	1	0
60–77	TP target	21	4	0	0	0	1	1	2	2	0	3	0	0	1	1	0	0	0	0	1	1	0	3	1	0
	TA filler	70	15	4	3	5	2	0	8	5	0	5	2	0	5	2	0	7	0	0	4	0	0	3	0	0

*TP, target present line-ups; TA, target absent line-ups. Post line-up confidence has been grouped into three categories: 0–60, 61–90, and 91–100%.*

**TABLE 4:** Confidence accuracy characteristic (CAC) analysis results in sequential line-ups per position and confidence category.

Age group	Position 1			Position 2			Position 3			Position 4			Position 5			Position 6			Position 7			Position 8		
	0–60	61–90	91–100	0–60	61–90	91–100	0–60	61–90	91–100	0–60	61–90	91–100	0–60	61–90	91–100	0–60	61–90	91–100	0–60	61–90	91–100	0–60	61–90	91–100
6–11	0.44	0.73	0.57	0.80	0.00	0.89	0.53	0.00	0.73	0.67	0.00	0.00	0.00	0.89	0.00	0.77	1.00	0.00	0.83	0.94	1.00	0.00	0.89	0.00
12–17	0.64	0.69	0.82	0.80	0.89	0.00	0.85	0.91	0.00	0.52	0.00	0.00	0.59	0.86	0.96	0.47	0.89	1.00	0.65	0.83	1.00	0.77	0.95	0.00
18–44	0.64	0.82	0.86	0.62	0.83	1.00	0.78	0.75	0.94	0.74	0.82	1.00	0.62	0.87	0.98	0.79	0.87	1.00	0.67	0.97	1.00	0.79	0.80	1.00
45–59	0.43	0.00	0.89	0.67	0.83	1.00	0.65	0.93	0.80	0.59	0.83	0.97	0.36	0.83	0.00	0.00	0.00	0.94	0.84	0.89	0.00	0.75	0.89	1.00
60–77	0.68	0.00	0.00	0.00	0.80	1.00	0.67	0.76	0.00	0.83	0.00	0.00	0.62	0.80	0.00	0.00	0.00	0.00	0.67	1.00	0.00	0.89	1.00	0.00

*CAC analysis calculation: target present target selections/[target present target selections + (target absent filler selections/line-up size: 8)]. Post line-up confidence has been grouped into three categories: 0–60, 61–90, and 91–100%.*

**TABLE 5:** Frequencies of TP target and TA filler selections in sequential line-ups per position and confidence category.

Age group	Selection	*N*	Position 1				Positions 2–8	
			0–60	61–90	91–100	0–60	61–90	91–100
6–11	TP target	42	3	4	2	16	11	6
	TA filler	135	30	12	12	58	14	9
12–17	TP target	68	7	3	4	26	22	6
	TA filler	165	31	11	7	83	26	7
18–44	TP target	165	12	10	4	73	45	21
	TA filler	372	53	18	5	228	66	2
45–59	TP target	53	2	0	2	22	18	9
	TA filler	149	21	8	2	86	27	5
60–77	TP target	21	4	0	0	10	6	1
	TA filler	70	15	4	3	37	11	0

*TP, target present line-ups; TA, target absent line-ups. Post line-up confidence has been grouped into three categories: 0–60, 61–90, and 91–100%.*

**TABLE 6:** Confidence accuracy characteristic analysis results in sequential line-ups per position and confidence category.

Age group	Position 1				Positions 2–8	
	0–60	61–90	91–100	0–60	61–90	91–100
6–11	0.44	0.73	0.57	0.69	0.86	0.84
12–17	0.64	0.69	0.82	0.71	0.87	0.87
18–44	0.64	0.82	0.86	0.72	0.85	0.99
45–59	0.43	0.00	0.89	0.67	0.84	0.94
60–77	0.68	0.00	0.00	0.68	0.81	1.00

*CAC analysis calculation: target present target selections/[target present target selections + (target absent filler selections/line-up size: 8)]. Post line-up confidence has been grouped into three categories: 0–60, 61–90, and 91–100%.*

The results from the CAC analyses revealed that as post decision confidence increased, so did accuracy in positions 2–8 for all age groups. This indicates that all age groups made more correct identifications when they were more confident. However, looking at the results from the first position, we see that confidence and accuracy were not clearly associated for all age groups. The confidence accuracy association is discernible for older children, young adults, and older adults. For young children, high confidence was less clearly associated with accuracy, which echoes earlier findings (Brackmann et al., 2019). However, the novelty of our findings is that the lack of association between confidence and accuracy in young children only appears to be true in the first line-up position. This indicates that in the first line-up position, young children were using a more liberal criterion, and in subsequent line-up positions, the criterion became more conservative, resembling the confidence accuracy relationship found in adults. It may be argued that due to a liberal response criterion, it is understandable that the first line-up position is over-sampled and more error prone, yet this does not explain why young children would adopt a more conservative criterion in later line-up positions. One possibility is that they are shifting their criterion and another is that a subsample of children has a tendency to select the first image in the line-up due to inhibition deficits, while those that can inhibit better and move on to the subsequent line-up images more closely resemble adults in their response bias.

For older children and older adults, the confidence accuracy relationship in the first line-up position resembled that of young adults, indicating no clear difference between the first line-up position and subsequent line-up positions. The oldest adults, on the other hand, tended to make high confidence errors in the first line-up position, indicating that (as with young children) the confidence accuracy relationship was not present in the first line-up position. Notably, all age groups except older adults showed an increased likelihood of selecting from the first position as distance increased, which is in line with the notion that a more liberal response bias increases first position selections (Wilson et al., 2019). The oldest adults were more likely to select an image from the first line-up position no matter the distance, indicating that although their overall response bias became more liberal as distance increased, their response bias in the first position was more liberal than in positions 2–8. This may indicate, as we have suggested for young children, that the difference between accuracies in line-up positions has to do with potential subgroups within the age category. Both young children and the oldest adults are selecting more from the first line-up position, but a more liberal response criterion does not appear to explain all the observed results. The fact that both age groups show a confidence accuracy association in line-up positions 2–8, but not in the first line-up position, indicates that there is some kind of bias in position 1 where high confidence is not well calibrated to accuracy. This may be due to some young children selecting due to low inhibition compared with other young children who do not have inhibition deficits (i.e., inhibition differences) and some older adults relying more on familiarity compared with other older adults who rely more on recollection (i.e., memory-related differences).

Combined, these results indicate that the first line-up position has different effects on different age groups and also impacts the confidence accuracy relationship. Notably, these results are based on a small number of observations because high confidence identifications (of either targets or fillers) were decreased with increased distance, and our interpretations are not based on the results of significance testing. Nevertheless, we have interpreted the results as indicating that for young children, misidentifications in the first line-up position may not only be associated with a liberal response bias but may reflect inhibition deficits. If not, it is difficult to understand how the confidence accuracy relationship can increase from the first line-up position to the extent that it resembles the confidence accuracy relationship seen in adults in line-up positions 2–8. Moreover, the lack of association between high confidence and accuracy for the oldest adults only applies to the first line-up position and also resembles the confidence accuracy relationship seen in adults in line-up positions 2–8. Here, also, individual memory-related differences may cause a subgroup to adopt a liberal response criterion making them more prone to select the first line-up position image, whereas those that move on the subsequent line-up positions are more conservative. This may explain the difference observed in the confidence accuracy relationship between the first and subsequent line-up position decisions. Here, we argue that a liberal response criterion is only a partial answer to age-related differences.

## Discussion

Photograph line-ups (i.e., photo arrays) are commonly used by the police (e.g., Wells, 1993; Wells and Olson, 2002). Although there is an ongoing debate regarding the superiority of either sequential or simultaneous line-ups (e.g., Wells et al., 2015), research on line-up position effects has not been very consistent, and within both sequential line-ups (e.g., Wilson et al., 2019) and simultaneous line-ups (Clark and Davey, 2005; Palmer et al., 2017; Meisters et al., 2018; Carlson et al., 2019), previous findings vary. Moreover, in eyewitness research, age comparisons are not very common and focused on young adults, with less focus on children, adolescents, and older adults (Erickson et al., 2015; Fitzgerald and Price, 2015; Brackmann et al., 2019). We were also unable to find an investigation of age and position effects, despite earlier findings showing that children and older adults tend to produce more false alarms in target absent line-ups (Bartlett and Memon, 2007; Bartlett, 2014; Fitzgerald and Price, 2015). This despite the evidence to suggest that there is reason to assume that there are age differences, as children and older adult memory strategies in eyewitness research have been suggested to rely on familiarity, whereas young adults (and perhaps adolescents) rely on recollection (Shing et al., 2008, 2010). Moreover, inhibitory control is still under development during childhood and adolescence (Nigg, 2000; Fitzgerald and Price, 2015), and old age gives rise to memory deficits (Shing et al., 2008), while inhibitory deficits in old age are less well established (Rey-Mermet and Gade, 2018). Last, based on findings that show that weaker facial encoding strength increases choosing bias (Mansour et al., 2012; Nyman et al., 2019b; Smith et al., 2019), there is also reason to assume that age and facial encoding strength may interactively affect line-up selection patterns.

In the present study, we investigated line-up position selections in both sequential and simultaneous target present and target absent line-ups by first showing four live targets to participants (one at a time) and asking them immediately after each viewing to take part in a line-up task. The targets presented to our participants were presented at distances between 5 and 110 m. The increased viewing distance can be seen as a proxy for facial encoding strength. In sequential line-ups, our main hypotheses were that: (1) inhibitory deficits in children and adolescents would manifest themselves by more indiscriminate selections in the first line-up positions compared with other age groups and other line-up positions 2–8, (2) increased distance (i.e., weaker facial encoding) would give rise to an increase in first line-up position selections for all age groups, but that the increase would be greatest for young children, adolescents, and the oldest adults. In simultaneous line-ups, our main hypothesis was that we would find a top row bias in our line-ups. However, due to the varying findings in the background literature, we also ran an exploratory analysis assuming that children and adolescents would show a tendency toward edge-aversion. Last, in both sequential and simultaneous line-ups, we ran exploratory analyses on the predictive value that line-up positions have on the likelihood of correct identifications in target present line-ups and false alarms in target absent line-ups.

### Line-Up Position Selections and Bias

In sequential line-ups, the results from our investigation of position effects in target present and target absent line-ups revealed that for all age groups, selections increase with increased distance and that selections of the first line-up position were more likely than selections of line-up positions 2–8. As distance increased, the likelihood of a first position selection increased dramatically. Moreover, in both target present and target absent line-ups, there was an interaction between age and position, and in target absent line-ups, there was also a main effect of age. The age differences showed that the likelihood of a first position image selection increased for all age groups in both target present and absent line-ups, except for the oldest adults whose first position selections appear to have stayed essentially the same with increased distance. The increase in first position selections was also steeper for young children and older children compared with young adults and older adults. These results are in line with notion that a more liberal response criterion results in a higher frequency of first line-up position selections in sequential line-ups (Wilson et al., 2019). However, our CAC analyses revealed that the response criterion was not kept constant between line-up positions and that young children and the oldest adults showed a more liberal criterion in the first line-up position compared with line-up positions 2–8, indicating a possible line-up position bias. The results may reflect that there are subgroups of young children who have inhibition deficits and a subgroup of older adults who rely more on familiarity compared with others in the same age category. Our interpretation of the results is that a liberal response criterion does not explain the whole picture, rather line-up positions have different effects on different age groups and that young children and the oldest adults are biased toward guessing more in the first line-up position compared with subsequent line-up positions. However, these findings are not based on significance testing, and more research is needed to substantiate this interpretation.

In simultaneous line-ups, we found that only in target absent line-ups was there a main effect of position, in slight favor of the top row selections. Although there was no effect in target present line-ups, the likelihoods appear to have been in slight favor of a top row bias in most age groups. However, the main effects of distance, age, and an interaction between distance and position that we found in target absent line-ups show that top or bottom row preference was moderated by both weaker encoding strength and based on the figures also by the age of the eyewitness. The exploratory analysis of central versus peripheral bias also revealed significant effects only in target absent line-ups. Here, the main effects of distance and age were moderated by an interaction between age and position, and an interaction between distance, age, and position on false alarm rates. The results indicate that central and peripheral line-up preferences are moderated by age and facial encoding strength. As distance increased, all age groups were more likely to select central images, except for young children where the opposite appears to have been the case. Young children were more likely to select from central line-up positions at a smaller distance, which supports our assumption that they show an edge-aversion, but this bias was reversed with increased distance, meaning that it is moderated by encoding strength. Moreover, as distance increased, so also did peripheral position bias for all age groups except older adults and the oldest adults, where the peripheral position bias decreased. This means that as facial encoding becomes weaker, older adults tend to show edge-aversion.

Combined, the results from the simultaneous line-ups suggest that biases occur in target absent line-ups but not target present line-ups. However, similar moderating effects of age and encoding strength were found in both target present and target absent line-ups. There was some support for a top row bias, but this was heavily influenced by age and increased distance, resulting in some cases in a bottom row bias. The same applied to central and peripheral bias, where increased distance reversed any preference in a certain age group. The implications are that central or peripheral or top or bottom row preference may be dependent on both age group and facial encoding strength, which also suggests that position effects in simultaneous line-ups are more complex than have previously been discussed in the literature and should be investigated further.

### Line-Up Position Effects on Hit and False Alarm Rates

In sequential line-ups, we found that selecting the first position in a target present line-up was less likely to lead to a correct identification (i.e., hit) compared with line-up positions 2–8. Although the difference dissipated with increased distance, the contrast is noticeable for all age groups at smaller distances. In target absent line-ups, we found no effects of line-up position on the likelihood of selecting the innocent suspect (i.e., false alarm). Looking at the results from both target present and target absent line-ups, the results indicate that the first line-up position has less diagnostic value, as the likelihood of a correct identification is significantly lower, while there is no difference in the likelihood of a false alarm. For example, at smaller distances, the likelihood of a young child making a correct identification is three times higher if they select from positions 2–8 instead of the first line-up position, while the likelihood of selecting the innocent suspect is the same.

In simultaneous line-ups, we found that at smaller distances, selecting from the top row in a target present line-up was more predictive (compared with the bottom row) of a correct identification. However, we also found that in target absent line-ups, top row selections were more likely to lead to an innocent suspect identification. The results indicate that selecting a line-up member from the top row is both more likely to lead to a correct identification (i.e., hit) and to an incorrect innocent suspect identification (false alarm). This most likely reflects the slight top row bias that we found. Next, when analyzing central versus peripheral position selections in simultaneous line-ups, we found that central position selections were more likely to lead to both a correct target present identification and a target absent incorrect innocent suspect identification. As with top versus bottom line-up selections, the central versus peripheral selections suggest that the slight bias toward central position selections inflates the chance of identifying the target and identifying an innocent person. Combined the results from simultaneous line-up position bias indicate that more research is needed to better understand how facial encoding strength affects position selections depending on the age of the eyewitness. It also implies that the notion of a clear-cut top, bottom, central, or peripheral line-up bias is a simplified and a reductionistic approach to the underlying causes of selection patterns.

### Practical Implications

The current study provides a systematic approach to investigating line-up position effects in target present and target absent simultaneous and sequential line-ups. We presented participants with real-life targets that were viewed under optimal conditions (i.e., no distractions and in daylight conditions), followed by an immediate line-up task. We also used viewing distance as a proxy for facial encoding strength, varying conditions from optimal viewing conditions (5 m) to sub-optimal viewing conditions (110 m). Our dataset also includes 1,588 participants ranging from 6 to 77 years of age, which enabled us to use multilevel analyses to analyze the complex relationship between eyewitness age, facial encoding strength, and line-up position selections. The main findings of the current study were that all age groups show an increased tendency toward first position selections in sequential line-ups when facial encoding strength became weaker (i.e., distance increased), and that younger children and the oldest adults showed a bias toward making more errors in the first versus subsequent line-up positions. In other words, the likelihood of an innocent suspect being chosen is higher if they are placed in the first line-up position of a sequential line-up. This problem may be counteracted if police recommendations state that a suspect should not be placed in the first position of a sequential line-up (e.g., Bill Blackwood Law Enforcement Management Institute of Texas, 2015); however, all police departments do not have such recommendations. Moreover, if the police avoid the first position and place the suspect in another position and if they also employ a line-up stopping rule (i.e., they discontinue the line-up once a selection is made), then the chances of selecting the suspect is greatly reduced for young children (6–11 years) or adults above 60 years. Last, our findings on the position effects in simultaneous line-ups suggest that position effects are moderated by age and facial encoding strength, which may be the reason that studies vary so much, meaning that more research is needed.

### Limitations

We have already discussed some of the limitations of our current design in an earlier publication (Nyman et al., 2019b); however, limitations that pertain to the current design are that there is evidence to suggest that in sequential line-ups, prior knowledge of the number of photos to be shown can result in an increase in choosing bias earlier on in the line-up (Horry et al., 2012; Carlson et al., 2016), but the same effect has been found when no information was given (Carlson et al., 2016; Meisters et al., 2018). As the present study is based on four trials per person, we could have purposefully neglected to mention the number of photos to be shown in the sequential line-ups, thus, enabling a comparison between the first trial where the participants were unaware versus the subsequent trials where they were aware of the number of photos to be shown. However, this would have created a difference between trials that may have proven detrimental to our overall goal of investigating the effects of distance and age on position effects. As was also discussed in our earlier publication (Nyman et al., 2019b), there were differences in accuracy rates between line-ups, which may have been due to differences in either line-up fairness or target distinctiveness. Nevertheless, as we have included targets as random factors in our statistical analyses, so that the effects are averaged across line-ups, we argue once again that line-up differences do not impact our current findings in any meaningful way.

## Conclusion

The current study was an attempt to systematically investigate line-up position effects by completely randomizing all image positions in target present and target absent sequential and simultaneous line-ups. Furthermore, we focused on two aspects that have not been combined with position effects before: facial encoding strength and eyewitness age. Our results indicate that the first line-up position in sequential line-ups is a perilous position to be placed in if you are an innocent suspect, and that depending on the age of the eyewitness and the strength of the facial encoding, the likelihood of misidentification is increased further. The tendency to select the first line-up position was also clearly prevalent in all age groups, but we also found that young children and the oldest show a tendency to be less well calibrated in the first line-up position compared with later line-up positions, indicating that they guessed and made errors even when confidence was high. This raises concerns regarding the use of sequential line-ups with these age groups. In simultaneous line-ups, there were tendencies toward position preferences, but these were tenuous and were moderated by distance and age, indicating that further research is needed to adequately address what might be the underlying causes of any selection preferences in simultaneous line-ups.

## Data Availability Statement

The original contributions presented in the study are publicly available. This data can be found here: https://osf.io/bqdmg/.

## Ethics Statement

The studies involving human participants were reviewed and approved by The Ethical Committee at Åbo Akademi University and the Ethical Committee at the Department of Psychology and Logopedics at Åbo Akademi University. Verbal and Electronic consent to participate in this study was provided by the participants’ legal guardian/next of kin.

## Author Contributions

TN, JA, JL, JK, and PS planned and designed the study. TN collected the data. TN, JA, JL, JK, and PS contributed to the analysis and interpretation of the data. TN wrote the first draft and completed the manuscript with the support of the other authors. All authors contributed to the article and approved the submitted version.

## Conflict of Interest

The authors declare that the research was conducted in the absence of any commercial or financial relationships that could be construed as a potential conflict of interest.
