# Supplementary material for: Line-Up Image Position in Simultaneous and Sequential Line-Ups: The Effects of Age and Viewing Distance on Selection Patterns
Source: Front Psychol. 2020 Jul 1;11:1349. doi: 10.3389/fpsyg.2020.01349 (PMC7342269; doi:10.3389/fpsyg.2020.01349)
Supplement: Supplementary file 1 [file Data_Sheet_1.PDF]

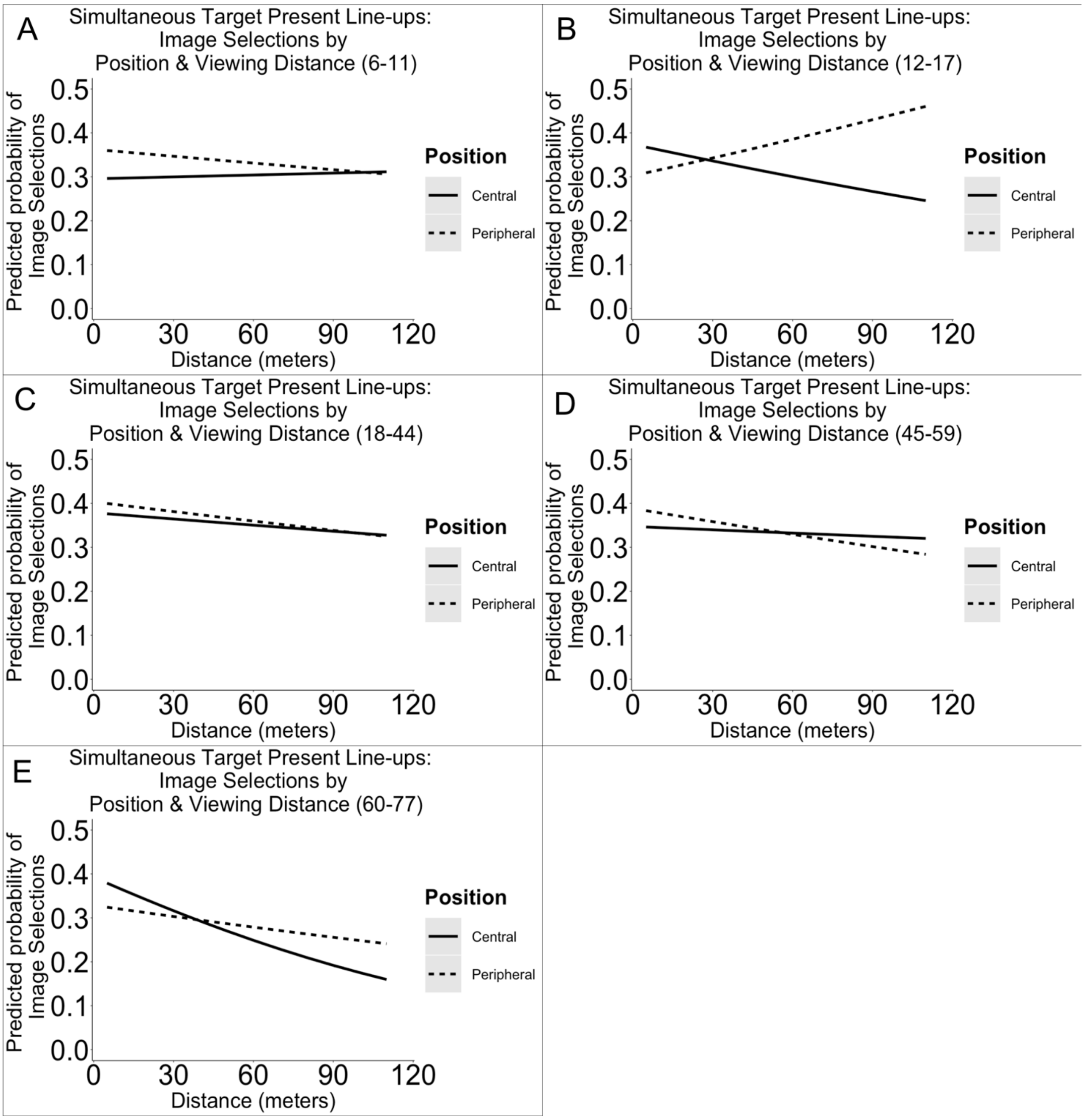

Figure 1. Illustration of the predicted probabilities of image selections in simultaneous target present line-ups per dichotomized line-up position (central versus peripheral), distance, and age group. Each panel represents the probabilities for a single age group: 6-11 **(A)**, 12-17 **(B)**, 18-44 **(C)**, 45-59 **(D)**, 60-77 (**E**).

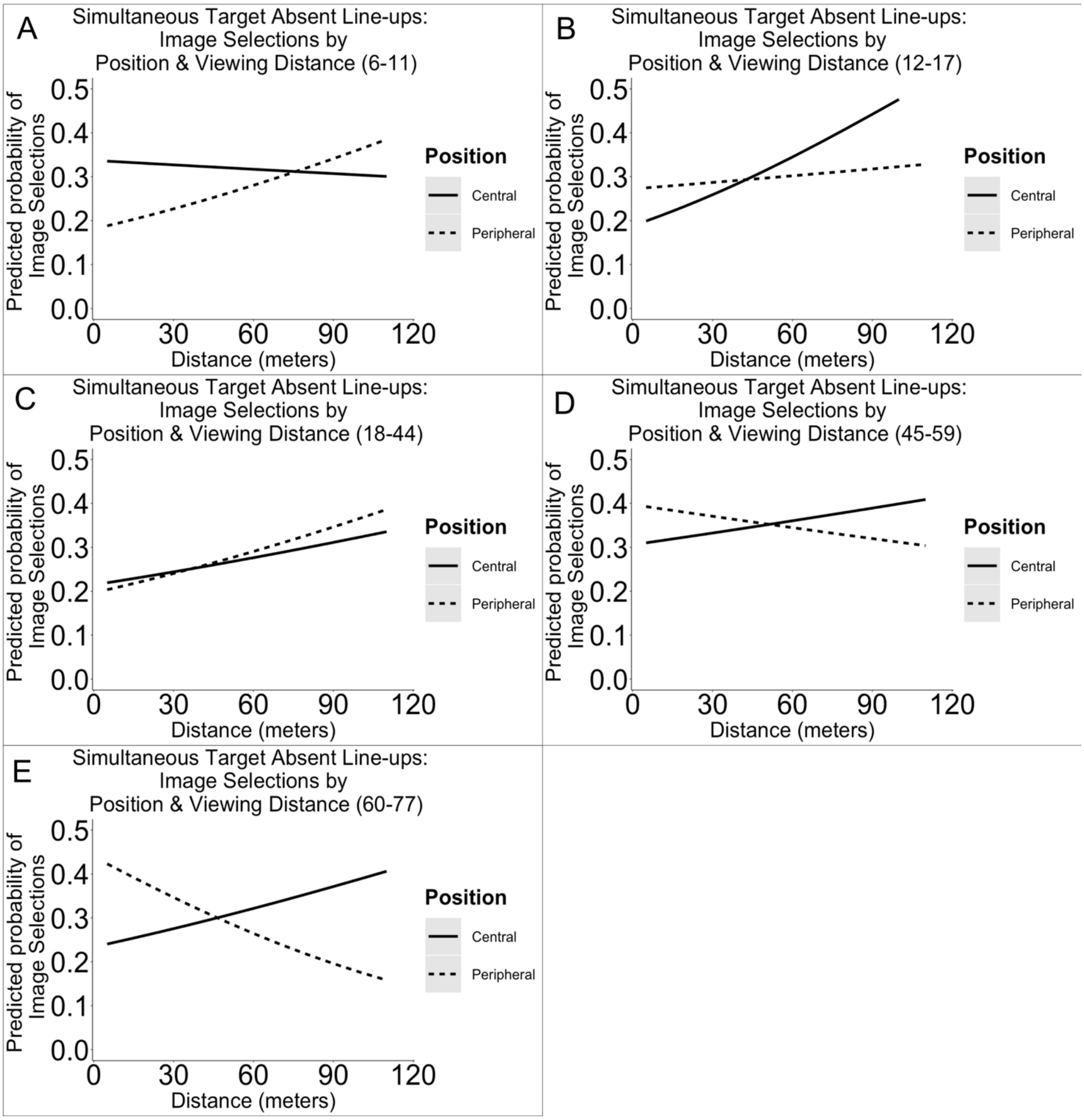

Figure 2. Illustration of the predicted probabilities of image selections in simultaneous target absent line-ups per dichotomized line-up position (central versus peripheral), distance, and age group. Each panel represents the probabilities for a single age group: 6-11 **(A)**, 12-17 **(B)**, 18-44 **(C)**, 45-59 **(D)**, 60-77 (**E**).

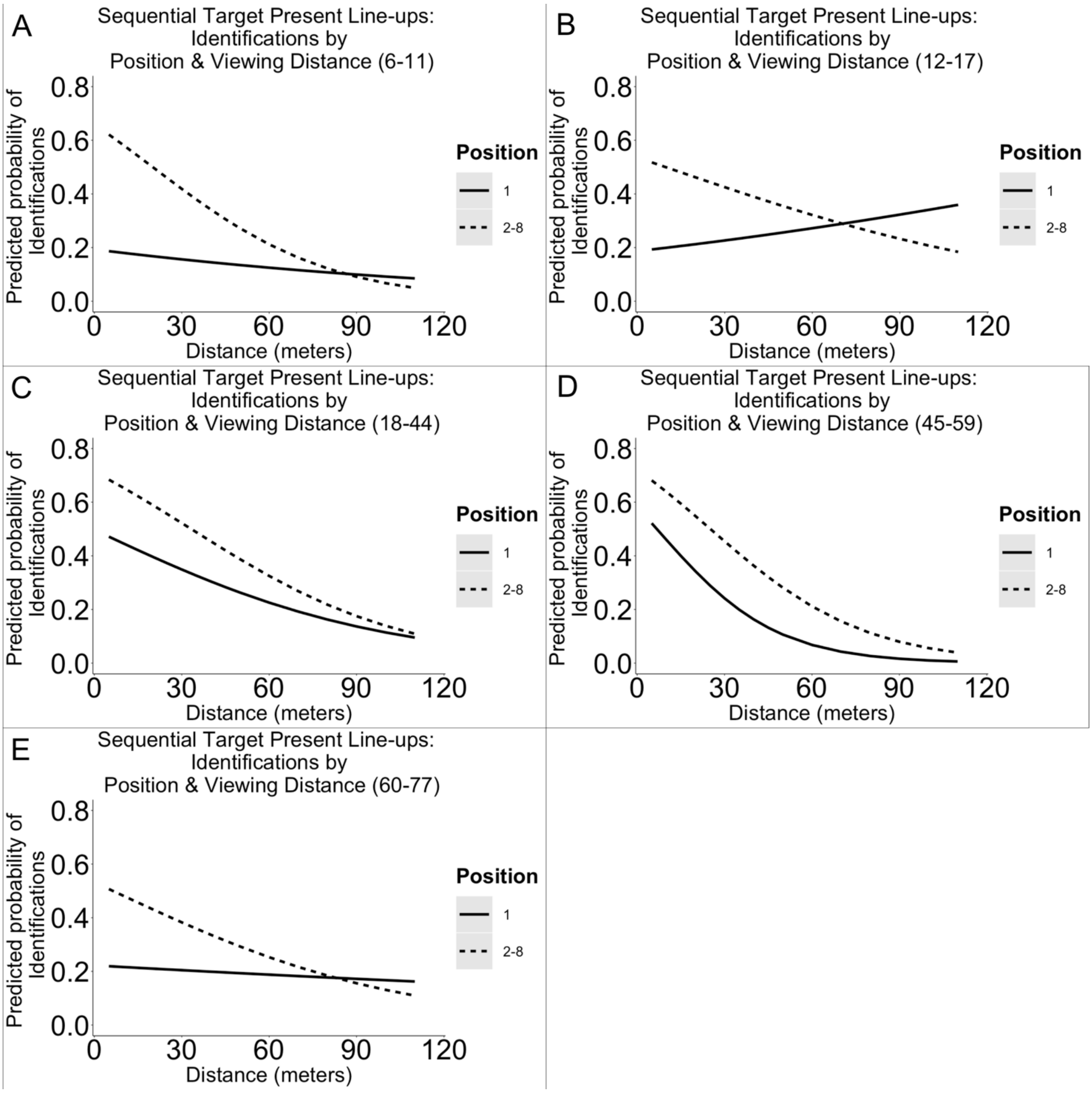

Figure 3. Illustration of the predicted probabilities of target identifications in sequential target present line-ups per dichotomized line-up position, distance, and age group. Each panel represents the probabilities for a single age group: 6-11 **(A)**, 12-17 **(B)**, 18-44 **(C)**, 45-59 **(D)**, 60-77 (**E)**.

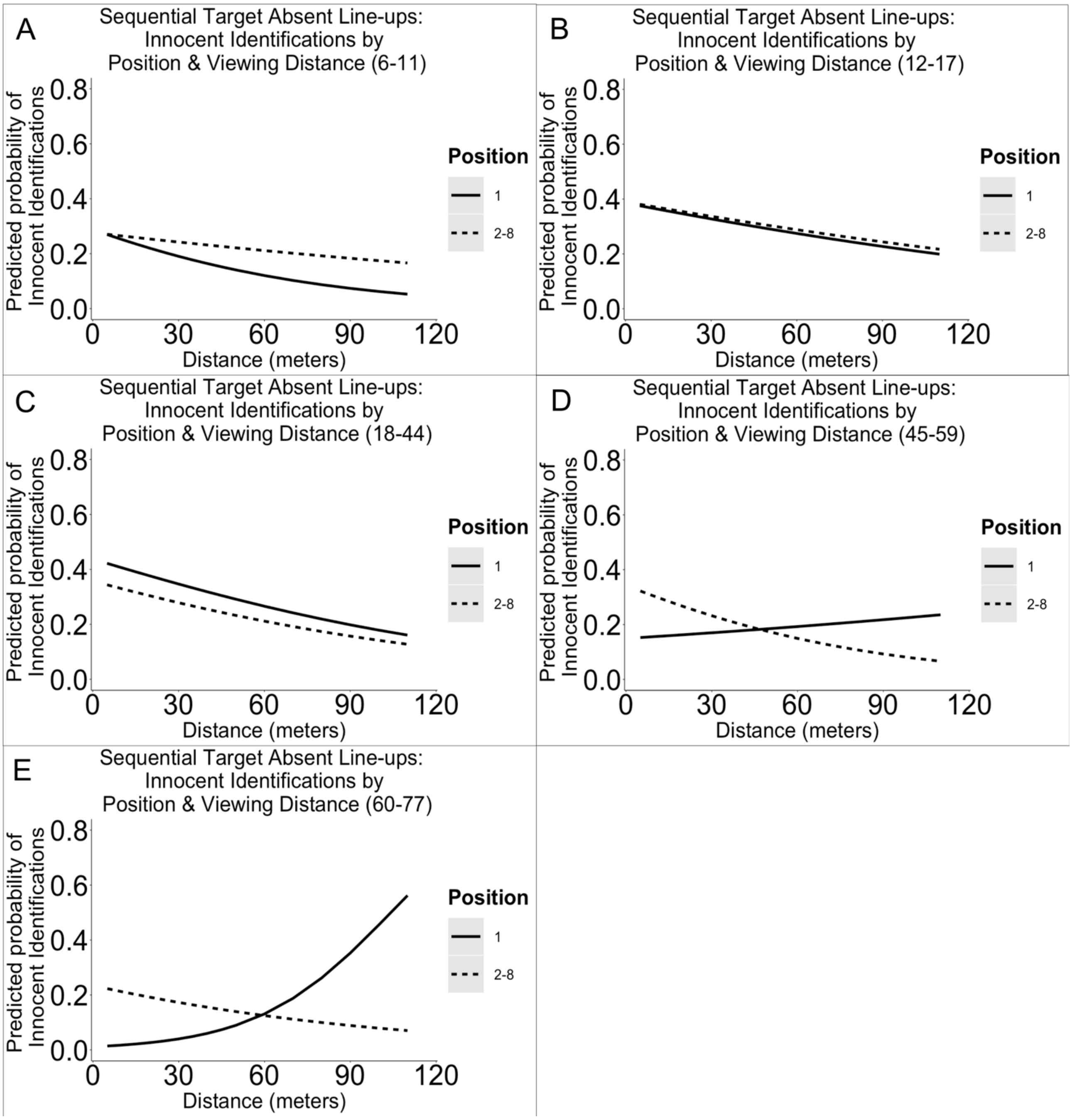

Figure 4. Illustration of the predicted probabilities of innocent identifications in sequential target absent line-ups per dichotomized line-up position, distance, and age group. Each panel represents the probabilities for a single age group: 6-11 **(A)**, 12-17 **(B)**, 18-44 **(C)**, 45-59 **(D)**, 60-77 (**E)**.

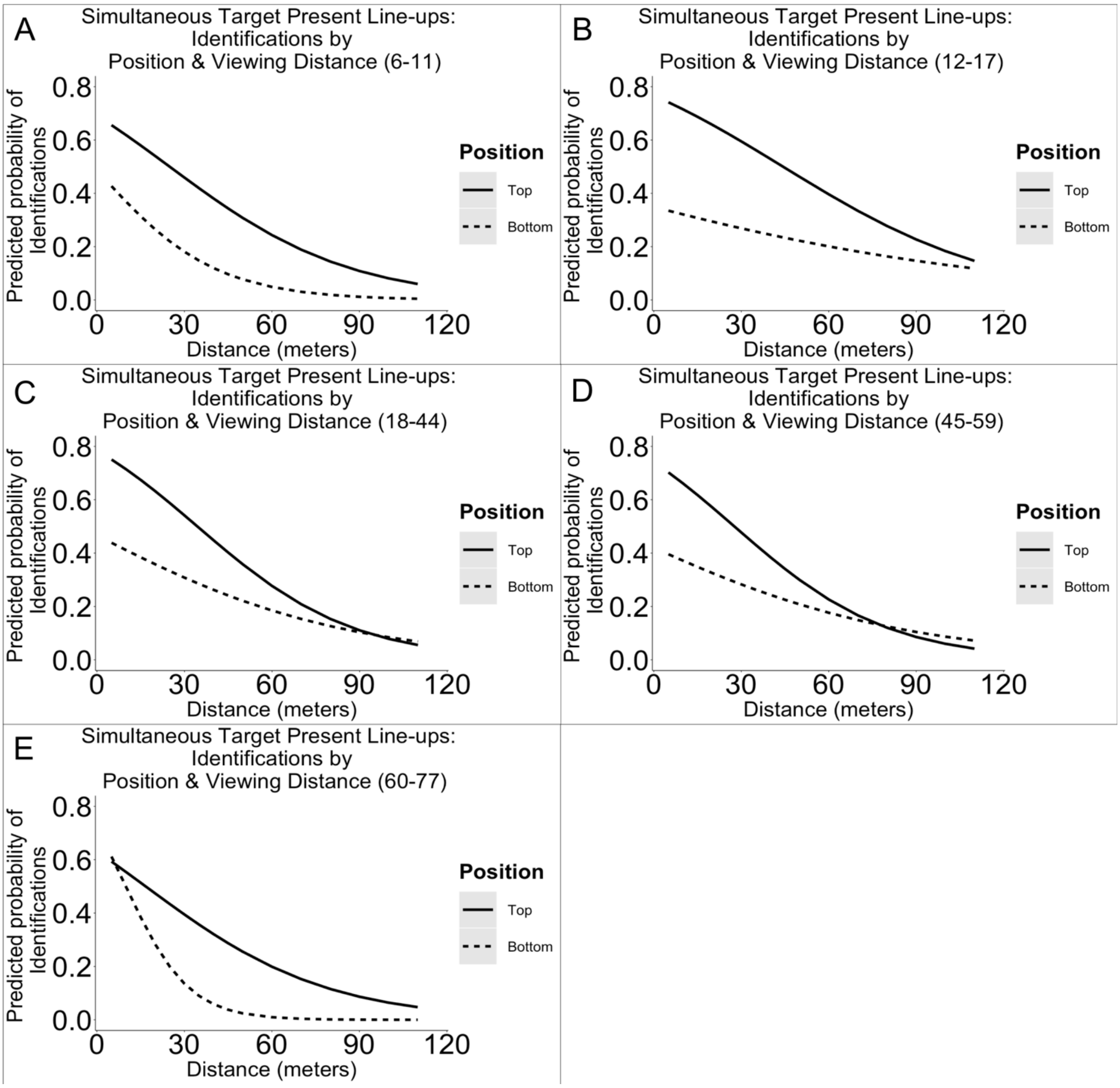

Figure 5. Illustration of the predicted probabilities of target identifications in simultaneous target present line-ups per dichotomized line-up position (top versus bottom), distance, and age group. Each panel represents the probabilities for a single age group: 6-11 **(A)**, 12-17 **(B)**, 18-44 **(C)**, 45-59 **(D)**, 60-77 (**E**).

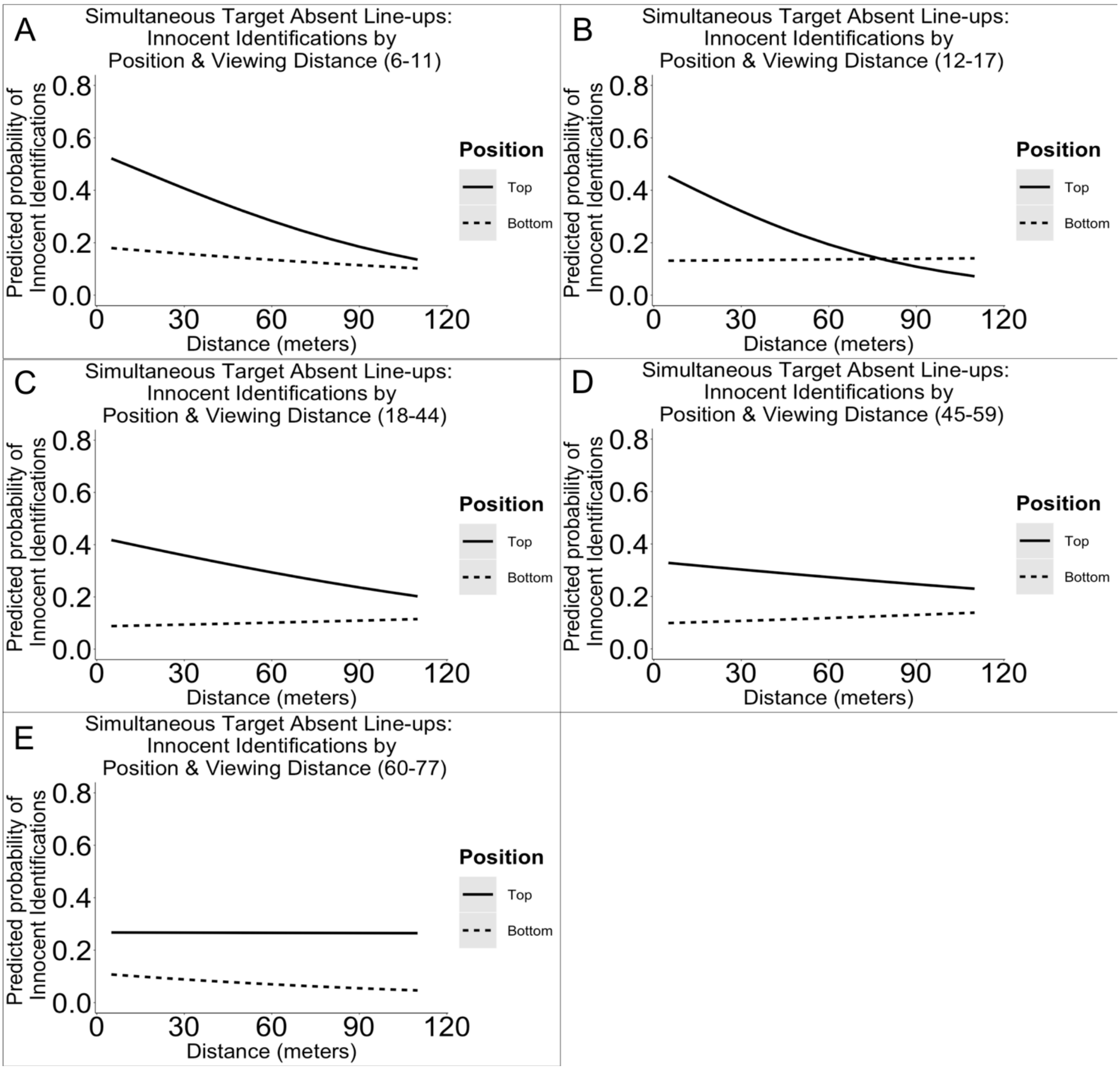

Figure 6. Illustration of the predicted probabilities of innocent identifications in simultaneous target absent line-ups per dichotomized line-up position (top versus bottom), distance, and age group. Each panel represents the probabilities for a single age group: 6-11 **(A)**, 12-17 **(B)**, 18-44 **(C)**, 45-59 **(D)**, 60-77 (**E**).

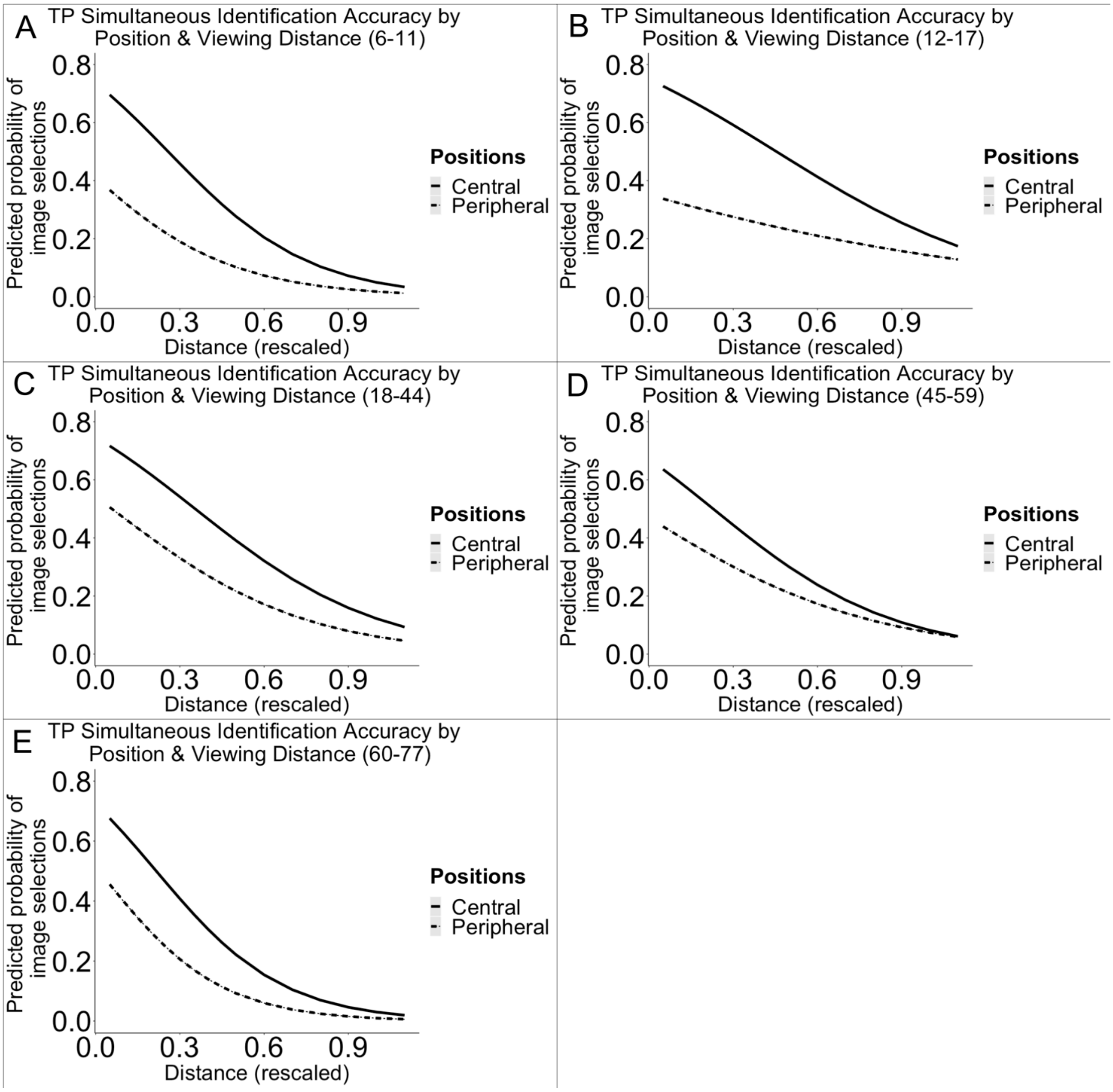

Figure 7. Illustration of the predicted probabilities of target identifications in simultaneous target present line-ups per dichotomized line-up position (central versus peripheral), distance, and age group. Each panel represents the probabilities for a single age group: 6-11 **(A)**, 12-17 **(B)**, 18-44 **(C)**, 45-59 **(D)**, 60-77 (**E)**.

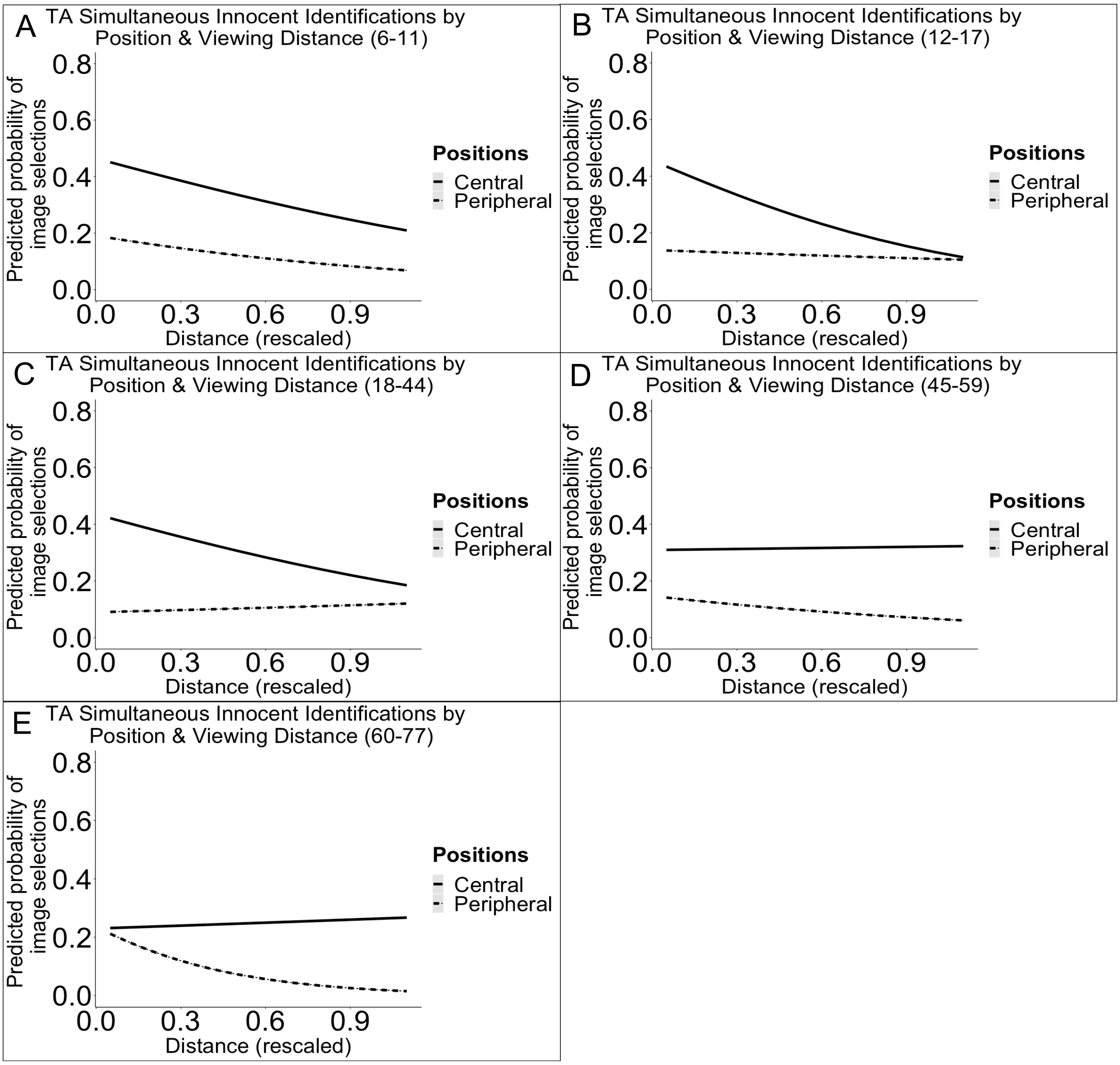

Figure 8. Illustration of the predicted probabilities of innocent identifications in simultaneous target absent line-ups per dichotomized line-up position (central versus peripheral), distance, and age group. Each panel represents the probabilities for a single age group: 6-11 **(A)**, 12-17 **(B)**, 18-44 **(C)**, 45-59 **(D)**, 60-77 **(E)**.
